# Factors Associated with Adiposity, Lipid Profile Disorders and the Metabolic Syndrome Occurrence in Premenopausal and Postmenopausal Women

**DOI:** 10.1371/journal.pone.0154511

**Published:** 2016-04-29

**Authors:** Edyta Suliga, Dorota Kozieł, Elżbieta Cieśla, Dorota Rębak, Stanisław Głuszek

**Affiliations:** 1 Department of the Prevention of Alimentary Tract Diseases, The Institute of Nursing and Midwifery, Faculty of Health Sciences, Jan Kochanowski University, Kielce, Poland; 2 Department of Surgery and Surgical Nursing with the Scientific Research Laboratory, The Institute of Medical Sciences, Faculty of Health Sciences, Jan Kochanowski University, Kielce, Poland; 3 Department of Developmental Age Research, Institute of Public Health, Faculty of Health Sciences, Jan Kochanowski University, Kielce, Poland; University of Padova, ITALY

## Abstract

The aim of the study was the assessment of the dependencies between a woman’s menopausal status and adiposity, lipid profile and metabolic syndrome occurrence, as well as finding out whether the correlations between the socio-demographic profile and lifestyle elements and adiposity, lipid profile and the risk of MetS are the same before and after menopause. A cross-sectional study was carried out on 3636 women, aged between 40–59, which involved a questionnaire interview, anthropometric measurements and fasting blood samples, on the basis of which the concentration of triglycerides, cholesterol and glucose was estimated. Before menopause, a greater adiposity (BMIβ = 0.08; %BFβ = 0.07; WCβ = 0.06) was characteristic for women living in a stable relationship than for single women. Women who smoked in the past were characterized by a higher BMI (β = 0.09) and WC (β = 0.06) in comparison with women who have never smoked, while after menopause a greater adiposity (%BFβ = 0.12) and a worse lipid profile (TCβ = 0.08; LDLβ = 0.07; HDLβ = -0.05; TGβ = 0.14) were present in women currently smoking, in comparison to women who have never smoked. After menopause, in women who had two or more children, a greater adiposity (BMIβ = 0.07 and 0.09; %BFβ = 0.05 and 0.07) and a higher risk of MetS (OR = 1.22, 95%CI: 1.03–1.44) was observed compared to nulliparous women, than before menopause. In women with a higher level of education, the risk of MetS after menopause was significantly lower compared with women with a lower level of education (OR = 0.74, 95%CI: 0.61–0.90). Physical activity after menopause had a higher influence on the decrease in the women’s adiposity (BMIβ = -0.11 v. -0.06; %BFβ = -0.11 v. -0.06; WCβ = -0.14 v. -0.08), than before menopause. In women not undergoing hormone replacement therapy, some of the socio-demographic factors and lifestyle elements affected adiposity, lipid profile and the risk of MetS differently before and after menopause, which requires verification through long-term research.

## Introduction

Menopause is a physiological phenomenon, defined as the final menstrual period and reflecting loss of the ovarian follicular function [1]. Natural menopause is recognized retrospectively after 12 months of amenorrhea. In white women from industrialized countries, it occurs, on average, at the age of 50–52, but the age of natural menopause may differ from 40 to 58 [1–3]. Most studies show that menopausal transformation in women leads to an increase of body mass and a change of adipose tissue distribution [4–6], deterioration of the lipid profile [7–9], and also to an increased frequency of menopausal syndrome (MetS) and its components [10, 11]. However, researchers do not agree whether greater adiposity and adverse metabolic changes should be explained to a greater extent by menopausal status and changes in the hormone balance related to it, or perhaps they are rather an independent effect of age [8, 12, 13]. Apart from age and menopausal status, socio-demographic factors such as parity, marital status, education, place of living, factors which modify women’s lifestyle, i.e. physical activity, dietary patterns, alcohol consumption and smoking may affect adiposity, lipid profile and the risk of MetS in perimenopausal women. According to some authors, greater parity was related to higher BMI values [14, 15] and a greater risk of the metabolic syndrome [16]. Trikudanathan et al. [13] did not find a significant correlation between a higher number of births and any of the adiposity indicators. Married women were characterized by a greater risk of obesity in comparison with unmarried women [17, 18], while the dependencies between marital status and the occurrence of the metabolic syndrome were unclear [11, 19, 20]. A lower risk of obesity, lipid disorders and MetS was related to a higher level of education [18, 19, 21–23]. Similarly, a lower occurrence of obesity and other risk factors of cardiovascular disease were more frequently found in a city environment than a rural one [24, 25]. Joshi et al. [26] and Sun et al. [27] obtained opposite correlations. Thus, the influence of the considered socio-demographic factors on adiposity, lipid profile and occurrence of the metabolic syndrome is not clear.

Menopause is an important event in a woman’s life, both in the aspect of health and from apsychological point of view. At this time of their lives, women experience not only biological changes, but also social and cultural ones. For many women menopause is a tangible marker of aging [28]. Research shows that postmenopausal women evaluate quality of life as worse compared to premenopausal women [29, 30]. The deficiency of estrogen after menopause is related to a decrease in energy expenditure [4, 31–33]. If there is no compensatory increase of physical activity and/or lower energy consumption, it leads to increased body mass. Changes in hormone concentration also cause metabolic changes in the body that appear at that time. A poorer quality of life, difficulties with maintaining previous body mass and the deterioration of metabolic indicators may induce postmenopausal women, especially those better educated, to introduce changes in their lifestyles aimed at decreasing the abovementioned problems. Therefore, we have formulated a hypothesis that socio-demographic variables and lifestyle elements may influence metabolic risk factors differently before and after menopause. To our knowledge so far, there have been no studies aimed at explaining this problem. Hence, the aim of the study was to determine the evaluation of correlations between women’s menopausal status and adiposity, lipid profile and the risk of metabolic syndrome and its components. Moreover, the purpose of the study was to find out which socio-demographic variables and lifestyle elements are related to excessive adiposity, an abnormal lipid profile and the risk of metabolic syndrome occurrence, and whether these dependencies are the same before and after menopause.

## Material and Methods

Research material was collected within the framework of the PONS project (POlish-Norwegian Study), which is prospective research on the health conditions of the inhabitants of the Świętokrzyskie Province in Poland. The study was approved by the Committee on Bioethics at the Faculty of Health Sciences, Jan Kochanowski University in Kielce. This approval refers only to the analysis of research results made available to us by the PONS team. The data, i.e. the questionnaire interviews, anthropometric measurements, and fasting blood samples, which were used to estimate the concentration of triglycerides, cholesterol and glucose, were collected as a part of the PONS project by a different team of researchers. Patients volunteered to participate in the study. The mandatory informed consent to participate in the study was collected in written form by the authors of the PONS project. The study was approved by the Ethics Committee within the Cancer Centre and Institute of Oncology in Warsaw, Poland. Detailed information regarding the project, group selection and research procedures were described in previously published papers [34, 35]. In short: all men and women aged between 45–64, permanently residing in the Kielecki Region in Poland, were invited to take part in the study. Voluntary participation rate in this age group was 12%. A small number of older people volunteered to take part in the study (65–66 years), and younger (37–44), in comparison with the previously intended age group. From all 13172 subjects (8725 women) aged between 37–66, a group of 5287 women aged between 40–59 was selected. Due to incomplete data, 969 female participants were rejected. Because of the strong influence of hormone replacement therapy (HRT) on the concentration of endogenous hormones and other biological markers, women who had ever used hormone therapy were excluded from the study (N = 682). Therefore, the further analysis involved 3636 women.

The research involved a questionnaire interview, anthropometric measurements and the analysis of fasting blood samples, on the basis of which the concentration of triglycerides, cholesterol and glucose was defined. In the questionnaire, the participants provided information related to socio-demographic data and lifestyle.

### Anthropometric measurements

The measurements of body weight and % of body fat were done by means of the body composition analyzer, Tanita SC 240 MA, with an accuracy of 0.1 kg and 0.1%. Body height measurements were done by means of the scale’s stadiometer, with an accuracy of 0.1 cm. The waist circumference was measured halfway between the lower rib edge and the upper iliac crest, by means of a metric measure with an accuracy of 0.1 cm.

### Assessment of blood pressure and biomarkers

Blood pressure was measured with the use of the blood pressure monitor Omron, model M3 Intellisense. The test was carried out on the artery of the right upper limb, when seated. In the study, the average of the two measurements was analysed. The glucose concentration in the blood serum was determined by means of the enzyme method with hexokinase, the concentration of triglycerides—by means of the phosphoglyceride oxidase-peroxidase method. The concentration of total cholesterol was determined by means of the enzyme method with esterase and cholesterol oxidase, and HDL and LDL cholesterol with the use of the colorimetric non-precipitation method.

### Lifestyle elements

The data related to the consumption of fruit and vegetables were collected by means of the FFQ questionnaire. PONS FFQ was constructed based on a previously developed and validated FFQ for the Poland branch of the PURE study and was characterized by good validity and reproducibility in relation to the referential method [36]. The questionnaire consisted of a list of standard size portion products. The participants of the study were asked about the frequency of consumption of certain portions of each product during the last year. The frequencies of consumption were classified as follows: less frequently than once a month or not at all, 1–3 times a month, once a week, 2–4 times a week, 5–6 times a week, once a day, 2–3 times a day, 4–5 times a day, 6 times a day or more, I don’t know, I refuse to answer the question. The answers related to the consumption frequency of some products from the questionnaire were transformed into daily consumption doses and then standardized by z-score. Alcohol consumption was evaluated on the basis of the frequency of alcoholic drinks consumption during the last 30 days in the following categories: every day, 4–5 times a week, 2–3 times a week, once a week, 2–3 times in the last 30 days, once during the last 30 days, not at all in the last 30 days, I don’t know, refusal. The consumption was transformed into the values of daily consumption doses and standardized by z-score. The respondents who smoked cigarettes on a daily basis during the study were classified as current smokers, and those who had not smoked for longer than 6 months—as former smokers, and the rest was regarded as nonsmokers. Physical activity was evaluated with the use of the International Physical Activity Questionnaire (IPAQ)–the long form. Total physical activity was calculated and expressed as metabolic equivalents (MET/min/week^-1^).

### Socio-demographic variables

The socio-demographic variables included: age (40–49; 50–59), place of residence (urban; rural), education (below a secondary level; secondary or higher—i.e. >12 years of education), parity (0, 1, 2, 3 and more children), marital status (married or in a stable relationship; single or a widow).

### Definitions of terms

The group of women after menopause included those with amenorrhea for at least 12 months (N = 2650). An appropriate body mass was a value of BMI <25.0 kg/m^2^, a precise amount of body fat was taken at the level of ≤35% BF [37]. The occurrence of metabolic syndrome was defined based on the recommendations of the International Diabetes Federation Task Force on Epidemiology and Prevention (joint interim statement in 2009) [38]. An appropriate total cholesterol value was <190mg/dL (<5mmol/L) and LDL-cholesterol <115mg/dL (<3mmol/L) [39].

### Statistical analysis

Distributions of the analysed characteristics were verified. For quantitative characteristics: age, BMI, %BF, waist circumference (WC), values of systolic and diastolic blood pressure, fasting blood sugar, total cholesterol (TC), HDL- and LDL-cholesterol and triglycerides (TG) values of mean, standard deviation, median and 95% CI and differences between the groups depending on menopausal status were calculated. In the case of qualitative data: place of residence, education, parity, marital status and menopausal status, the structure indicators were estimated. To test the structural equation index (qualitative data), a chi-squared test was used, in the case of quantitative data, the significance of differences was estimated by means of the Student-T Test. In order to evaluate the influence of particular socio-demographic factors and lifestyle elements on adiposity, lipid profile and the metabolic syndrome components in two groups of menopausal status separately, multiple regression analysis was applied along with backward elimination procedure. The abovementioned research procedure was applied twice. For the entire study group (aged between 40–59) and for the most narrow age group, for which, due to multiplicity, such analysis was possible (48–52). In each model the following variables were included in the 0–1 system: place of residence (0—city; 1—country), age (0–40–49; 1–50–59); education (0 –lower than secondary,1 –secondary and higher, i.e. >12 years of education), marital status (0 –single; 1 –married/in a stable relationship), parity (0 –without children; 1—respectively: one child, two children, three children and more), smoking (0-nonsmokers; 1-former smokers;1-current smokers), for the consumption of fruit and vegetables, alcohol and physical activity, a reference level (0) was established at the level of the lowest tertile. Adiposity indicators, lipid profile and the components of metabolic syndrome were standardized into a mean and standard deviation of all subjects and standardized β coefficients are presented in this paper. This procedure enabled us to compare the effect of individual variables (of different measurement units) on the values of the analyzed characteristics in the standard deviation units. Moreover, in the whole group the interactions between menopausal status and each factor and result included in the analysis were testedwith the use of MANOVA/MANCOVA analysis.

The OR and 95% CI values were calculated in two groups of menopausal status, allowing for socio-economic predictors and lifestyle elements in this model (fruit, vegetable and alcohol consumption and physical activity). In the analysis procedure, only those factors which showed a significant correlation with MetS were included in the model. The other factors, due to the lack of a significant correlation, were not taken into consideration. The reference levels were taken as follows: for menopausal status: the group before menopause, for age: the younger age group (40–49), for the place of residence: a city, for parity: lack of children, for marital status: single. In the case of smoking—non-smoking women, while in relation to the consumption of fruit and vegetables, alcohol and physical activity, the reference point involved the values of the first, lowest tertile (T1). The p values p<0.05 were considered statistically significant. All material was devised with the use of the statistical package Statistica 10.0.

## Results

Postmenopausal women were found to be significantly older, they were more often the residents of cities and more often lived alone compared to women before menopause (Table 1). No significant differences were found in the level of education and parity depending on menopausal status. Women after menopause drank less alcohol and there were more former smokers among them. However, there were differences in fruit and vegetable consumption as well as physical activity, depending on menopausal status. The analysis of the metabolic risk factors revealed that women after menopause were characterized by greater adiposity, worse lipid profile and a more frequent MetS occurrence and its components compared to women before menopause.

**Table 1:** Differences in anthropometric and biological parameters and socio-demographic variables and lifestyle between pre- and postmenopausal women.

Variables	Premenopausal women (N = 1316) X±SD; Me (95% CI)	Postmenopausal women (N = 2320) X±SD; Me (95% CI)	P
Age (years)	49.7±3.1; 50.0 (45.0–56.0)	55.2±3.0; 56.0 (48.0–59.0)	0.001^a^
Place of living: urban (%)	751 (57.07)	1430 (61.64)	0.010^b^
Education: secondary or higher(%)	1014 (77.05)	1732 (74.26)	NS
Parity (%)			NS
one child	212 (16.11)	351 (15.13)	
two children	621 (47.19)	1075 (46.34)	
three and more	386 (29.33)	704 (30.34)	
Marital status: in a stable relationship (%)	1052 (79.94)	1785 (76.94)	0.050^b^
Fruit and vegetables (servings/day)	4.06±1.71; 3.85 (1.34–8.00)	4.07±1.71; 3.91 (1.31–7.93)	NS
Smoking (%)			0.001^b^
former smokers	315 (23.94)	730 (31.47)	
current smokers	250 (19.00)	473 (20.39)	
Alcohol consumption (servings/day)	0.06±0.09; 0.03 (0.00–0.36)	0.05±0.08;0.03 (0.00–0.36)	0.001^a^
Total activity (MET-/min/week^-1^)	2502.1±1332.5; 2325.0 (552.0–5118.0)	2458.5±1243.5 2238.0 (576–5022.0)	NS
BMI (kg/m^2^)	26.9±4.7; 26.2 (20.1–38.3)	28.1±4.9; 27.4 (20.6–39.7)	0.001^a^
% BF	34.1±6.7; 34.5 (20.2–46.1)	36.2±6.5; 36.6 (22.6–47.4)	0.001^a^
Waist circumference (cm)	85.1±11.2; 84.0 (67.0–111.0)	88.3±11.5; 87.0 (69.0–114.0)	0.001^a^
Total cholesterol (mg/dL)	205.4±33.0; 204.0(146.0–276.0)	217.3±38.8; 215.0 (146.0–296.0)	0.001^a^
LDL-cholesterol (mg/dL)	121.6±29.2; 120.0 (69.0–182.2)	132.2±34.7; 131.0 (69.2–201.5)	0.001^a^
HDL-cholesterol (mg/dL)	63.7±14.7; 63.0 (38.0–96.0)	62.5±14.3; 61.0 (39.0–95.0)	0.050^a^
Triglycerides (mg/dL)	100.8±53.6; 88.0 (39.0–243.0)	113.0±53.9; 100.0 (43.0–255.0)	0.001^a^
Glucose (mg/dL)	92.0±14.7; 90.0 (73.0–121.0)	95.8±19.4; 93.0 (75.0–133.0)	0.001^a^
Hypertension (%)	726 (55.17)	1401 (60.39)	0.010^b^
Metabolic syndrome (%)	538 (40.88)	1234 (53.19)	0.000^b^

^a^—Student-T Test;

^b^—chi-squared test;

NS- non significant; BMI—Body Mass Index; BF- Body Fat; LDL—Low Density Lipoproteins; HDL- High Density Lipoproteins

In the analysis carried out separately for two groups of menopausal status, the most important factors significantly related to higher values of all analyzed adiposity factors, both before and after menopause were: the older age of the female participants, living in a rural environment, a low level of education, the lowest physical activity (T1 vs. T3) and the lowest alcohol consumption (T1 vs. T3) (Table 2). However, standardized values of beta coefficients show that an independent differentiating effect of the education level was stronger after menopause than before (-0.14 v. -0.10 for BMI and -0.13 v. 0.08 for %BF). Before menopause, women living in a stable relationship were characterized by significantly higher values of all adiposity indicators, whereas after menopause, having a stable partner was only related to a greater adipose tissue, while BMI and waist circumference were not significantly correlated with a marital status. The conducted analysis revealed the presence of interactions between parity and menopausal status. Parous women, before menopause had lower, and after menopause—higher values of adiposity indicators, compared tonulliparous women. The interactions were also present between menopausal status and smoking. Before menopause, a higher BMI and waist circumferences were found in former smokers, whereas current smokers were characterized by less adipose tissue, compared to women who had never smoked. After menopause greater adipose tissue was present in former smokers but no significant correlations were observed with BMI and waist circumference. Lower BMI and waist circumferences were found in current smokers. The dependencies between physical activity and adiposity indicators turned out to be stronger in postmenopausal women (-0.11 v. -0.06 for %BF and -0.14 v. -0.08 for WC). Moreover, after menopause less adipose tissue was found in women not only in the third, but also in the second tertile of physical activity.

**Table 2:** The influence of the socio-demographic factors and lifestyle elements on adiposity indicators in women between 40–59 before and after menopause—the results of multiple regression analysis by backward elimination.

Menopausal status		Predictors	β (β standard error)	p
BMI	pre	Age	0.15(0.03)	0.05
		Place of living	0.07(0.03)	0.001
		Education	-0.10(0.03)	0.001
		Marital status	0.08(0.03)	0.01
		One child	-0.07(0.03)	0.01
		Two children*	-0.09(0.03)	0.001
		Former smokers	0.09(0.03)	0.01
		T3 of alcohol consumption	-0.07(0.03)	0.01
		T3 of PA	-0.06(0.03)	0.001
	po	Age	0.04(0.02)	0.01
		Place of living	0.08(0.02)	0.001
		Education	-0.14(0.02)	0.05
		Two children	0.07(0.03)	0.001
		≥3 children	0.09(0.03)	0.01
		Current smokers*	-0.14(0.02)	0.05
		T3 of alcohol consumption	-0.06(0.02)	0.001
		T2 of PA	-0.07(0.02)	0.01
		T3 of PA	-0.11(0.02)	0.050
% BF	pre	Age	0.15(0.03)	0.001
		Place of living	0.07(0.03)	0.05
		Education	-0.08(0.03)	0.01
		Marital status	0.07(0.03)	0.05
		≥3children	-0.06(0.03)	0.05
		Current smokers*	-0.12(0.03)	0.001
		T3 of alcohol consumption	-0.07(0.03)	0.05
		T3 of PA	-0.06(0.03)	0.05
	po	Age	0.08(0.02)	0.001
		Place of living	0.07(0.02)	0.01
		Education	-0.13(0.02)	0.001
		Marital status	0.05(0.02)	0.05
		Two children	0.05(0.03)	0.05
		3≥children	0.07(0.03)	0.05
		Former smokers*	0.12(0.02)	0.001
		T2 of PA	-0.08(0.02)	0.01
		T3 of PA	-0.11(0.02)	0.001
WC	pre	Age	0.15(0.03)	0.01
		Place of living	0.11(0.03)	0.05
		Education	-0.15(0.03)	0.05
		Marital status	0.06(0.03)	0.001
		One child*	-0.09(0.03)	0.001
		Two children*	-0.11(0.03)	0.001
		Former smokers	0.06(0.03)	0.05
		T3 of alcohol consumption	-0.06(0.03)	0.01
		T3 of PA	-0.08(0.03)	0.001
	po	Age	0.07(0.02)	0.001
		Place of living	0.12(0.22)	0.01
		Education	-0.16(0.02)	0.01
		Child*	0.08(0.03)	0.001
		Two children*	0.12(0.04)	0.01
		≥3 children	0.17(0.04)	0.01
		Current smokers	-0.08(0.02)	0.001
		T3 of alcohol consumption	-0.06(0.02)	0.001
		T3 of PA	-0.07(0.02)	0.001
		T3 of PA	-0.14(0.02)	0.001

pre-premenopausal status; po-postmenopausal status; BMI- Body Mass Index; BF- Body Fat; WC- Waist Circumference; PA-physical activity; T—tertile

*—Interaction with menopausal status (p≤0.05)

A worse lipid profile, both before and after menopause, was most significantly correlated with the age of the female participants, and in case of triglycerides, also withresiding in the countryside and currently smoking (Table 3). Additionally, a lower HDL concentration was related to a poor education and low alcohol consumption (T1 v. T2 before menopause and T1 v. T3 after menopause). Before menopause, a significant correlation was found between total cholesterol concentration and a high consumption of vegetables. Moreover, a higher triglyceride concentration was related to a lower level of education, whereas a lower concentration—with higher alcohol consumption. After menopause, generally, a worse lipid profile was characteristic of current smokers. What is more, parous women, compared to nulliparous had a lower HDL concentration, and a similar dependency before menopausewas present only in women who had given birth to at least 3 children. A higher HDL concentration after menopause was noted in women from the highest physical activity tertile (T3 v. T1).

**Table 3:** The influence of the socio-demographic factors and lifestyle elements on lipid profile in women between 40–59 before and after menopause—the results of multiple regression analysis by backward elimination.

Menopausal status		Predictors	β(β standard error)	p
TC	pre	Age	0.07(0.03)	0.050
		T2 of fruit and vegetables	0.06(0.03)	0.010
	po	Age	0.05(0.02)	0.050
		Current smokers	0.08(0.02)	0.001
LDL	pre	Age	0.06 (0.02)	0.050
	po	Age	0.06(0.02)	0.010
		Current smokers	0.07(0.02)	0.010
HDL	pre	Age	-0.07(0.0)	0.010
		Level of education	0.08(0.03)	0.010
		≥3children	-0.08(0.03)	0.010
		T2 of alcohol consumption	0.10(0.03)	0.010
	po	Education	0.08(0.02)	0.001
		Child	-0.10(0.03)	0.010
		Two children	-0.14(0.04)	0.001
		≥3children	-0.12(0.04)	0.010
		Current smokers	-0.05(0.02)	0.050
		T3 of alcohol consumption	0.12(0.02)	0.001
		T3 of PA	0.056(0.02)	0.010
TG	pre	Age	0.17(0.028)	0.001
		Place of living	0.08(0.03)	0.010
		Current smokers	0.08(0.03)	0.010
	po	Place of living	0.07(0.02)	0.010
		Education	-0.07(0.02)	0.010
		Current smokers	0.14(0.02)	0.001
		T3 of alcohol consumption	-0.05(0.02)	0.050

pre-premenopausal status; po-postmenopausal status; TC- Total Cholesterol; LDL—Low Density Lipoproteins; HDL- High Density Lipoproteins; TG-Triglycerides; T—Tertile; PA-Physical Activity

Age was correlated with a higher glucose concentration both before menopause and after (Table 4). Additionally, before menopause a higher glucose concentration was found in women who gave birth to 1–2 children, current smokers and those who consumed most alcohol (T3 v. T1). After menopause, a higher glucose concentration was only present in the female participants with poorer education. Elevated systolic pressure, both before and after menopause, was characteristic of older women, whereas in the case of diastolic blood pressure, a significant dependency was observed only before menopause. Moreover, before menopause higher blood pressure was present in nulliparous women compared to those who had 2 children. After menopause, a higher blood pressure was found in poorer educated women.

**Table 4:** The influence of the socio-demographic factors and lifestyle elements on MetS components in women between 40–59 before and after menopause—the results of multiple regression analysis by backward elimination.

Menopausal status		Predictors	β(β standard error)	p
Glucose	pre	Age	0.11(0.03)	0.001
		One child*	0.06(0.03)	0.05
		Two children*	0.09(0.03)	0.01
		Current smokers	0.07(0.03)	0.01
		T3 of alcohol consumption	0.07(0.03)	0.05
	po	Age	0.05(0.02)	0.05
		Education	-0.05(0.02)	0.05
Systolic blood pressure	pre	Age*	0.14(0.02)	0.001
	po	Age*	0.09(0.03)	0.001
		Education*	-0.11(0.02)	0.001
Diastolic blood pressure	pre	Age	0.10(0.03)	0.001
		Two children	-0.06(0.03)	0.05
	po	Place of living	0.06(0.02)	0.05
		Education	-0.010(0.02)	0.001

pre-premenopausal status; po-postmenopausal status; T—Tertile

*—Interaction with menopausal status (p≤0.05)

A higher risk of MetS, both before and after menopause, was present in older women and current smokers (Table 5). After menopause a greater risk of MetS occurred also in women with a lower level of education and those who had given birth to 2 children, compared to nulliparous women.

**Table 5:** Odds ratio for the metabolic syndrome in women aged between 40–59 and 48–52 before and after menopause.

Menopausal status		Predictors	OR (95%CI)	p
Age 40–59	pre	Age	1.76(1.41–2.18)	0.001
		Current smokers	1.34(1.01–1.77)	0.05
	po	Age	2.08(1.38–3.16)	0.001
		Education	0.74(0.61–0.90)	0.01
		Two children	1.22(1.03–1.44)	0.05
		Current smokers	1.37(1.12–1.69)	0.05
Age 48–52	pre	Age	1.49(1.09–2.03)	0.05
		Current smokers	1.70(1.16–2.49)	0.01
	po	Place of living	1.61(1.07–2.43)	0.05
		Current smokers	0.56(0.35–0.89)	0.05

pre-premenopausal status; po-postmenopausal status

In order to restrict the effect of age on the considered dependencies, an additional analysis was conducted on a limited age range (48–52 years of age). However, even in such a narrow 5-year group, age was found to be statistically significantly differentiating the adiposity indicators, lipid profile and the risk of MetS (Tables 5–7). The results of this analysis generally confirmed previously obtained results, i.e. the occurrence of significant interactions between menopausal status and smoking as well as differentiating influence of parity on the adiposity indicators and certain MetS components. What is more, they revealed the correlation between menopausal status and the consumption of fruit and vegetables in case of glucose concentration and also a significant correlation between a low level of education and certain MetS components, i.e. higher triglycerides concentration and higher blood pressure in postmenopausal women.

**Table 6:** The influence of the socio-demographic factors and lifestyle elements on adiposity indicators in women between 48–52 before and after menopause—the results of multiple regression analysis by backward elimination.

Menopausal status		Predictors	β(β standard error)	p
BMI	pre	Age	0.07(0.03)	0.05
		Place of living	0.11(0.04)	0.05
		Marital status	0.08(0.04)	0.05
		One child	-0.10(0.04)	0.01
		Two children	-0.15(0.04)	0.05
		Former smokers*	0.14(0.04)	0.05
		T3 of alcohol consumption	-0.10(0.04)	0.01
		T3 of PA	-0.08(0.04)	0.05
	po	Place of living	0.12(0.04)	0.001
		Education	-0.14(0.04)	0.001
		≥3children	0.09(0.04)	0.05
		Current smokers*	-0.20(0.04)	0.001
		T3 of alcohol consumption	-0.09(0.04)	0.05
		T3 of PA	-0.08(0.04)	0.05
%BF	pre	Age	0.11(0.04)	0.01
		Place of living	0.11(0.04)	0.01
		Marital status	-0.10(0.04)	0.01
		One child	-0.08(0.04)	0.05
		Two children	-0.12(0.04)	0.001
		Former smokers*	0.18(0.03)	0.001
		T3 of alcohol consumption	-0.09(0.04)	0.05
	po	Age	0.09(0.04)	0.05
		Place of living	0.09(0.04)	0.05
		Education	-0.17(0.04)	0.001
		Marital status	-0.10(0.04)	0.01
		Current smokers*	-0.17(0.04)	0.05
WC	pre	Age	0.09(0.04)	0.01
		Place of living	0.12(0.04)	0.01
		Education	-0.10(0.04)	0.01
		≥3children	0.17(0.04)	0.001
		Former smokers	0.07(0.04)	0.05
		T3 of alcohol consumption	-0.08(0.03)	0.05
		T3 of PA	-0.09(0.04)	0.05
	po	Age	0.08(0.04)	0.05
		Place of living	0.13(0.04)	0.01
		Education	-0.20(0.04)	0.001
		Marital status	0.09(0.04)	0.05
		Current smokers	-0.13(0.04)	0.01

pre-premenopausal status; po-postmenopausal status; T—tertile; PA-physical activity; BMI- Body Mass Index; BF- Body Fat; WC- Waist Circumference

*—Interaction with menopausal status (p≤0.05)

**Table 7:** The influence of the socio-demographic factors and lifestyle elements on MetS components in women between 48–52 before and after menopause—the results of multiple regression analysis by backward elimination.

Menopausal status		Predictors	β(beta standard error)	p
Glucose	pre	Former smokers*	0.09(0.04)	0.05
		T3 of fruit and vegetables*	0.08(0.04)	0.05
		T3 of PA	-0.08(0.04)	0.05
	po	T3 of fruit and vegetables*	-0.11(0.04)	0.001
Systolic blood pressure	pre	Age	0.08(0.04)	0.05
	po	Education	-0.10(0.04)	0.05
		T3 of PA	-0.09(0.04)	0.05
Diastolic blood pressure	pre	Two children	-0.09(0.04)	0.01
		Former smokers	-0.07(0.04)	0.05
	po	Education	-0.16(0.04)	0.001
		Two children	0.09(0.04)	0.05

pre-premenopausal status; po-postmenopausal status; T-tertile; PA—physical activity

*—Interaction with menopausal status (p≤0.05)

## Discussion

Regardless of menopausal status, greater adiposity, less beneficial lipid profile and a greater risk of MetS and some of its components were mainly correlated with the older age of the female participants. Moreover, greater adiposity was characteristic of women from a rural environment, with a lower level of education, low physical activity and the lowest alcohol consumption. Current smoking was related to a worse lipid profile and a higher risk of the MetS occurrence and some of its components. However, some of the analyzed socio-demographic factors and lifestyle elements were related to adiposity and metabolic factors of women differently before and after menopause.

The results of the studies conducted up to now, indicate that menopausal status and age may be independent predictors of the cardiovascular disease and MetS risk factors [10, 11, 40, 41]. Lovejoy et al. [4] have shown that in middle-aged women there is a growth of subcutaneous adipose tissue along with age, and menopause *per se* was related to the increase of total and visceral adipose tissue. The results of the studies done up to now also indicate that menopausal status and age are independent predictors of cardiovascular risk factors and MetS [10, 11, 40, 41]. The longitudinal study also showed that the MetS frequency increases during the perimenopausal period and in the first years following menopause, independent of growing old and other risk factors of cardiovascular disease, such as body weight gain and smoking [42]. Dasgupta et al. [12] have reported that menopausal status and obesity related to it, is the main risk factor of metabolic aberrations during menopause, more important than age. Trikudanathan et al. [13] obtained opposite results. They proved that although women after menopause had higher BMI values, visceral subcutaneous adipose tissue, this dependency was mostly the effect of age. Chae and Derby [43] show that proatherogenic changes in the lipid profile and apolipoprotein seem to be related particularly to the ovaries aging, while adverse changes of other risk factors of cardiovascular disease may be under a greater influence of chronological aging. In this study, the strong influence of age was additionally confirmed in the analysis conducted on a limited age range (48–52 years of age), in which a differentiating effect of age was observed even overa period of 5 years.

A greater risk of obesity, lipid disorders and MetS found in individuals with a lower level of education, was shown in several populations [18, 19, 22, 23, 44, 45]. The research conducted by Rohrman et al. [46], showed that although during observation, body mass gain was observed in all education levels, the gain was the lowest in men and women with a higher education (average difference body mass gain in the group with a high vs. low education level was respectively 120 g annually and 70 g annually). The occurrence of similar correlations was confirmed by several other authors [47, 48]. These authors also suggest that BMI differences between individuals with higher and lower education levels, getting greater with time, probably increase. It is possible that health in older age, more than in younger age, depends on our conscious action. This fact may be explained by the stronger influence of education level reported in this study on health indicators in postmenopausal women than on premenopausal ones and constitutea definite verification of the hypothesis constructed by us.

The place of residence is in many countries a factor significantly differentiating health indicators [24, 25, 27, 49–51]. It effects, among others, health behavior, access to health service, living and working conditions. In developed countries a lower intensity of obesity occurrence and other risk factors of cardiovascular disease was usually found in city environment [24, 25, 51]. The results of the conducted studies confirmed a greater risk of excessive body mass and higher TG concentration, and at the age between 48–52, a greater risk of MetS among rural women.

Research done by other authors showed a greater risk of obesity among married women in comparison to unmarried ones [17, 18]. This study has also shown that having a stable partner is associated withgreater values of adiposity indicators, especially in women before menopause. As Averett et al. [21] claim, single women show greater concern about their looks: being slim is commonly thought to be more attractive. Women who do not care about attracting a partner, may allow themselves to gain body weight. It is possible that this phenomenon is stronger in younger women, before menopause, while after menopause, other factors, not related to marital status, start to contribute to body mass and waist circumference. The influence of marital status on metabolic syndromes was found to be insignificant, which is confirmed by the study results obtained by other authors. Al-Daghri et al. [19] have shown that the correlation between marital status and the risk of MetS was only significant for men, not for women. Ben Ali et al. [11] also did not find any significant differences in the MetS risk, comparing single women and married ones.

The results of this study showed that before menopause women who had given birth to 1 or 2 children had a lower BMI and waist circumferences, whereas after menopause women who had 2 and more children were characterized by higher adiposity indicators, and those aged between 48–52 also by greater risk of MetS. Although Trikudanathan et al. [13] did not find any correlation between the number of childbirths and any adiposity indicators, many authors in their studies have shown that a higher number of childbirths was connected with higher BMI values [14, 15, 52] and a higher risk of MetS [16, 53]. Gunderson et al. [54], in a prospective study found that childbirth was directly related to MetS occurrence in women with gestational diabetes. A higher glucose concentration noted in women before menopause, who have given birth 1–2 children, may to some extent confirm the existence of such a correlation. It is a well known fact that pregnancy causes considerable changes in the risk factors related to cardiovascular diseases, such as accumulation of central abdominal fat, atherogenic lipid profile and insulin resistance [55, 56]. Research has also showed that in the longer perspective, after the reproductive period is over, different obesity indicators increase along with the number of children [15, 57]. However Bobrow et al. [52] confirmed that at every parity level, average BMI values were lower in women who have breastfed, compared to those who have not. Breastfeeding, involving large energy expenditures, may lead to a decreasing body mass and metabolism regulation after the childbirth, which can be observed in premenopausal women. However, it seems possible that after a longer period, and also due to hormonal changes related to menopause, benefits resulting from breastfeeding disappear, causing higher adiposity values and the risk of MetS.

Lower adiposity of current smokers and higher—of former smokers compared to non-smokers confirmed by us, is in compliance with the results obtained by other authors, suggesting that smoking can be related to a lower BMI [58, 59], while giving up smoking with an increased BMI [59, 60]. The influence of smoking on adiposity indicators varies, depending on the menopausal status of the female participants. Lower BMI and WC in current smokers, occurring after menopause, can be an effect of older age, so a longer period of smoking and thus a longer period of nicotine influence on the body. Greater BMI and WC of former smokers, compared to non-smokers before menopause, may result from the fact that premenopausal women were generally younger and the time since they stopped smoking could have been shorter. It has been demonstrated that the greatest body mass gain occurs within the first year of smoking cessation and is much slower in the next years [58]. The results of our study also confirm a positive correlation between current smokers and an increased risk of abnormal lipid profile and a MetS occurrence, which was also observed in several other studies [22, 61]. According to Slagter et al. [61], an increased risk of MetS is independent of BMI and results mainly from a lower HDL cholesterol concentration, a bigger waist circumference with the BMI under control and a higher TG concentration.

We did not observe that greater fruit and vegetable consumption decreased the risk of excessive adiposity, abnormal lipid profile and MetS, despite the fact that a former study showed a significant correlation between dietary patterns and metabolic obesity with normal body mass in this population [35]. It was only concluded that higher fruit and vegetable consumption before menopause was connected with a higher total cholesterol concentration, and in the age group 48–52 with a higher glucose concentration, and after menopause—with its lower concentration. Due to a cross-sectional nature of the study, it may only indicate a higher consumption of this group of products by patients before menopause with an increased concentration of cholesterol and glucose, which after menopause could have led to a desired effect in the form of a lower glucose concentration.

Higher alcohol consumption, among study participants both before and after menopause, was related to a lower adiposity, higher HDL concentration and a lower triglycerides concentration after menopause, but higher glucose concentration—before menopause. Results obtained by other authors confirm that moderate alcohol consumption may protect against increased body mass gain [62], increase the concentration of HDL-cholesterol [63] and decrease the risk of some MetS components in women [22]. However, it should be noted that the declared alcohol consumption, even by women who were in the third, highest tertile, was very low, and on average equaled 2.32g ethanol a day.

Low physical activity is a well documented risk factor of overweight and obesity, abnormal lipid profile and the occurrence of MetS [22, 64–67]. Moreover, some research indicates that physical activity may be a stronger preventative measure from MetS than reducing the consumption of calories [67]. Our study confirmed the protective role of physical activity in relation to excessive adiposity and some of the MetS components, i.e. abdominal obesity, abnormal HDLand glucose but only in the age range between 48–52. It is characteristic that in postmenopausal women adiposity was lower not only in the third, the highest tertile of physical activity, but also in the second tertile, compared to the first one. A prospective study carried out by Kushi et al. [68], revealed that in postmenopausal women taking moderate physical activity only once a week, provided considerable health benefits. Also a cohort study of women aged 45 and older confirmed that even 1 hour of walking per week was related to a lower risk of ischemic heart disease in comparison to a total lack of activity [69]. The results of the study confirm that after menopause, even a little physical activity decreases the risk of abnormal health indicators.

### Study limitations

The main limitation of this study is the cross-sectional nature of the research, therefore all lifestyle changes that might have been made by the subjects, may influence the possibility of a precise explanation of the causal link betweenlifestyle elements and metabolic health indicators. Another limitation was including the level of education in the analysis as an indicator of socio-economic position, and not income. However, we assumed that although the level of education does not always adequately reflect the current financial situation of an individual, in most cases it reflects his or her social status and ability to gain and use knowledge, influences starting and maintaining a healthy lifestyle. A strong aspect of this paper is the large number and relatively narrow age bracket of the study participants (40–59). In the majority of studies of this type, a much broader age bracket is taken into consideration, which may affect the analyzed dependencies through secular changes.

## Conclusions

The factor which was most significantly related to greater adiposity, worse lipid profile and the risk of MetS, regardless of menopausal status of the female participants, was age. Moreover, higher adiposity was characteristic of women from a rural environment, with a lower level of education, low physical activity and lowest alcohol consumption. A worse lipid profile as well as a higher risk of MetS and some of its components, were related to current smoking. Certain of the considered socio-demographic factors, such as: parity, marital status, education and lifestyle elements (smoking, physical activity) were correlated with women’s adiposity and metabolic indicators differently before and after menopause, which confirms our hypothesis. Before menopause, women living in a stable relationship were characterized by a higher adiposity compared to single women, whereas after menopause the independent effect of this factor turned out to be insignificant. Female former smokers had a higher BMI and waist circumferences before menopause compared to women who have never smoked, while after menopause greater adiposity and worse lipid profile were present in current smokers compared to women who have never smoked. After menopause, in women who had 2 and 3 and more children, compared to nulliparous women, higher adiposity and a greater risk of MetS than before menopause were confirmed. Education was significantly more related to adiposity after menopause than before. Also the risk of MetS was significantly lower in better educated women comparedto poorer educated ones. Although no significant differences were found in the physical activity of the subject women depending on menopausal status, after menopause this variable had a significantly stronger influence on the decrease of adiposity in more active women, than before menopause.

Concluding, it should be stated that in women who did not use hormonal replacement therapy, some of the socio-demographic factors, as well as lifestyle elements were related to adiposity and metabolic indicators in a different way before and after menopause, which however, requires corroboration through long-term studies. More precise knowledge concerning the effect of these factors will allow us to identify groups of higher metabolic risk earlier and to take more effective preventive measures, allowing for women’s menopausal status. Because of this, it can contribute to a further decrease in cardiovascular and neoplasm prevalence and mortality.
